# Hexakis(1*H*-imidazole-κ*N* ^3^)mangan­ese(II) triaqua­tris(1*H*-imidazole-κ*N* ^3^)manganese(II) bis­(naphthalene-1,4-dicarboxyl­ate)

**DOI:** 10.1107/S1600536808011677

**Published:** 2008-04-26

**Authors:** Jun-Hua Li, Jing-Jing Nie, Duan-Jun Xu

**Affiliations:** a Department of Chemistry, Zhejiang University, People’s Republic of China

## Abstract

In the crystal structure of the title compound, [Mn(C_3_H_4_N_2_)_6_][Mn(C_3_H_4_N_2_)_3_(H_2_O)_3_](C_12_H_6_O_4_)_2_, there are uncoordinated naphthalene­dicarboxyl­ate dianions and two kinds of Mn^II^ complex cations, both assuming a distorted octa­hedral geometry. One Mn^II^ cation is located on an inversion center and is coordinated by six imidazole mol­ecules, while the other Mn^II^ cation is located on a twofold rotation axis and is coordinated by three water mol­ecules and three imidazole units. The naphthalene­dicarboxyl­ate dianions are linked to both Mn^II^ complex cations *via* O—H⋯O and N—H⋯O hydrogen bonding, but no π–π stacking is observed between aromatic rings in the crystal structure.

## Related literature

For general background, see: Su & Xu (2004 ▶); Liu *et al.* (2004 ▶). For a related structure, see: Derissen *et al.* (1979 ▶).
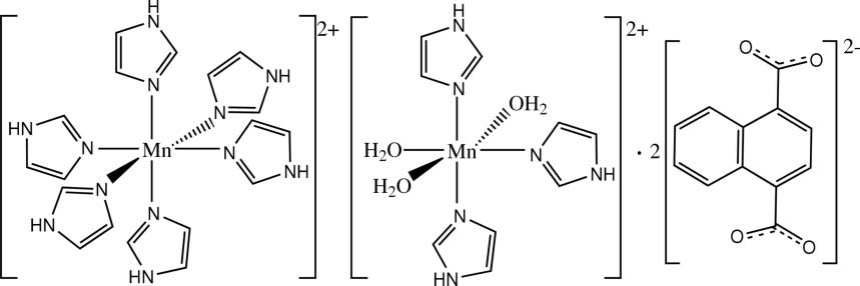


## Experimental

### 

#### Crystal data

[Mn(C_3_H_4_N_2_)_6_][Mn(C_3_H_4_N_2_)_3_(H_2_O)_3_](C_12_H_6_O_4_)_2_
*M* *_r_* = 1205.0
Orthorhombic, 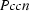

*a* = 29.605 (4) Å
*b* = 9.4619 (12) Å
*c* = 20.534 (3) Å
*V* = 5752.0 (14) Å^3^
*Z* = 4
Mo *K*α radiation
μ = 0.51 mm^−1^
*T* = 295 (2) K
0.33 × 0.30 × 0.18 mm

#### Data collection

Rigaku R-AXIS RAPID IP diffractometer
Absorption correction: multi-scan (*ABSCOR*; Higashi, 1995 ▶) *T*_min_ = 0.790, *T*_max_ = 0.912
61105 measured reflections
5131 independent reflections
4174 reflections with *I* > 2σ(*I*)
*R*_int_ = 0.047

#### Refinement

*R*[*F* ^2^ > 2σ(*F* ^2^)] = 0.049
*wR*(*F* ^2^) = 0.135
*S* = 1.07
5131 reflections
367 parameters
5 restraints
H-atom parameters constrained
Δρ_max_ = 1.06 e Å^−3^
Δρ_min_ = −0.66 e Å^−3^

### 

Data collection: *PROCESS-AUTO* (Rigaku, 1998 ▶); cell refinement: *PROCESS-AUTO*; data reduction: *CrystalStructure* (Rigaku/MSC, 2002 ▶); program(s) used to solve structure: *SIR92* (Altomare *et al.*, 1993 ▶); program(s) used to refine structure: *SHELXL97* (Sheldrick, 2008 ▶); molecular graphics: *ORTEP-3 for Windows* (Farrugia, 1997 ▶); software used to prepare material for publication: *WinGX* (Farrugia, 1999 ▶).

## Supplementary Material

Crystal structure: contains datablocks I, global. DOI: 10.1107/S1600536808011677/sg2237sup1.cif

Structure factors: contains datablocks I. DOI: 10.1107/S1600536808011677/sg2237Isup2.hkl

Additional supplementary materials:  crystallographic information; 3D view; checkCIF report

## Figures and Tables

**Table 1:** Selected bond lengths (Å)

Mn1—N1	2.250 (3)
Mn1—N3	2.271 (2)
Mn1—N5	2.276 (2)
Mn2—N7	2.283 (3)
Mn2—N9	2.190 (4)
Mn2—O1*W*	2.265 (2)
Mn2—O2*W*	2.129 (3)

**Table 2:** Hydrogen-bond geometry (Å, °)

*D*—H⋯*A*	*D*—H	H⋯*A*	*D*⋯*A*	*D*—H⋯*A*
O1*W*—H1*A*⋯O4	0.93	1.85	2.772 (3)	170
O1*W*—H1*B*⋯O1^i^	0.86	2.02	2.875 (3)	175
O2*W*—H2*A*⋯O3^ii^	0.85	1.78	2.624 (3)	172
N2—H2*N*⋯O4	0.86	1.87	2.730 (4)	176
N4—H4*N*⋯O2^iii^	0.86	1.91	2.765 (3)	177
N6—H6*N*⋯O2^iv^	0.86	1.95	2.810 (4)	178
N8—H8*N*⋯O1^v^	0.86	2.02	2.870 (4)	167
N10—H10*A*⋯O3^vi^	0.86	1.78	2.560 (7)	150

Symmetry codes: (i) 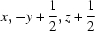; (ii) 
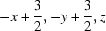
; (iii) 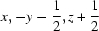; (iv) 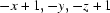; (v) 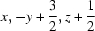; (vi) 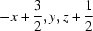.

**Fig. 1.**
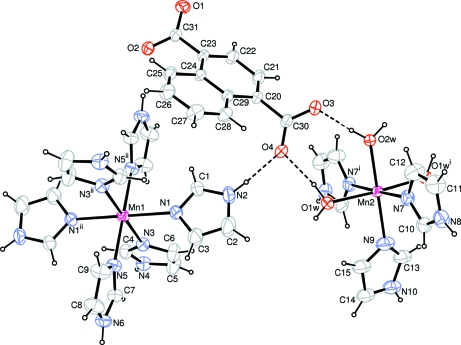
The molecular structure of the title compound with 30% probability displacement (arbitrary spheres for H atoms). One of the disordered imidazole components is omitted for clarify. Dashed lines indicate hydrogen bonding [symmetry codes: (i) -x + 3/2, -y + 3/2, z; (ii) -x + 3/2, -y + 1/2, z + 1].

## supplementary crystallographic information

### Comment

As part of our ongoing investigation on the nature of π-π stacking (Su & Xu, 2004; Liu *et al.*, 2004), the title compound incorporating naphthalenedicarboxylate has recently been prepared and its crystal structure is reported here.

The crystal consists of uncoordinated naphthalenedicarboxylate dianions and two kinds of Mn^II^ complex cations. Both Mn^II^ complex cations assume distorted octahedral geometry (Table 1). The Mn1 is located on an inversion center and coordinated by six imidazole molecules, while the Mn2 is located about a twofold rotation axis and is coordinated by three water and three imidazole molecules (Fig. 1). The O2 (water) and N9 atoms are also located about the twofold axis, but the disordered N9-imidazole ring does not lie on the twofold axis. The naphthalenedicarboxylate dianion is not coordinated to the Mn^II^ cation but is linked to both Mn^II^ complex cations *via* O—H···O and N—H···O hydrogen bonding (Fig. 1 and Table 2). No π-π stacking is observed between adjacent naphthalene rings. Two carboxyl groups are twisted with respect to the naphthalene ring system with dihedral angles of 52.5 (3)° and 48.7 (3)°, which are larger than those found in the structure of free naphthalenedicarboxylic acid (*ca* 40°; Derissen *et al.*, 1979).

### Experimental

An aqueous solution (10 ml) containing naphthalene-1,4-dicarboxyllic acid (0.108 g, 0.5 mmol) and sodium carbonate (0.053 g, 0.5 mmol) was refluxed for 0.5 h, then tetraaqua-manganese dichloride (0.099 g, 0.5 mmol) and imidazole (0.136 g, 2 mmol) were added to the above solution. After cooling to room temperature the solution was filtered. The single crystals of the title compound were obtained from the filtrate after 1 d.

### Refinement

The N9-containing imidazole is disordered over  two sites, both close to a twofold rotation axis, and was refined with half site occupancy, while the N9 atom is located on the twofold axis but not disordered. In the structure refinement, the coordinates of the N9 atom located on the twofold axis were refined by introducing an artificial bias of 0.02 (in fraction) to its *x* and *y* parameters, after several cycles of refinement the coordinates of the N9 atom shifted to the initial special position of (3/4, 3/4, 0.64847). Bond distances for the disordered imidazole were restrained. Water H atoms were located in a difference Fourier map and refined as riding in as-found relative positions with *U*_iso_(H) = 1.5*U*_eq_(O). Aromatic H atoms were placed in calculated positions with C—H = 0.93 Å and N—H = 0.86 Å, and refined in riding mode with *U*_iso_(H) = 1.2*U*_eq_(C,*N*). The highest peak in the final difference Fourier map is apart from the N9 atom by 0.03 Å.

### Crystal data

**Table:**

[Mn(C_3_H_4_N_2_)_6_][Mn(C_3_H_4_N_2_)_3_(H_2_O_1_)_3_](C_12_H_6_O_4_)_2_	*F*_000_ = 2496
*M**_r_* = 1205.0	*D*_x_ = 1.391 Mg m^−^^3^
Orthorhombic, *P**c**c**n*	Mo *K*α radiation λ = 0.71073 Å
Hall symbol: -P 2ab 2ac	Cell parameters from 6856 reflections
*a* = 29.605 (4) Å	θ = 3.0–24.0º
*b* = 9.4619 (12) Å	µ = 0.51 mm^−^^1^
*c* = 20.534 (3) Å	*T* = 295 (2) K
*V* = 5752.0 (14) Å^3^	Prism, yellow
*Z* = 4	0.33 × 0.30 × 0.18 mm

### Data collection

**Table:**

Rigaku R-AXIS RAPID IP diffractometer	5131 independent reflections
Radiation source: fine-focus sealed tube	4174 reflections with *I* > 2σ(*I*)
Monochromator: graphite	*R*_int_ = 0.047
Detector resolution: 10.0 pixels mm^-1^	θ_max_ = 25.2º
*T* = 295(2) K	θ_min_ = 1.4º
ω scans	*h* = −35→35
Absorption correction: multi-scan(ABSCOR; Higashi, 1995)	*k* = −11→10
*T*_min_ = 0.790, *T*_max_ = 0.912	*l* = −24→24
61105 measured reflections	

### Refinement

**Table:**

Refinement on *F*^2^	Secondary atom site location: difference Fourier map
Least-squares matrix: full	Hydrogen site location: inferred from neighbouring sites
*R*[*F*^2^ > 2σ(*F*^2^)] = 0.049	H-atom parameters constrained
*wR*(*F*^2^) = 0.135	*w* = 1/[σ^2^(*F*_o_^2^) + (0.0611*P*)^2^ + 4.7866*P*] where *P* = (*F*_o_^2^ + 2*F*_c_^2^)/3
*S* = 1.07	(Δ/σ)_max_ = 0.001
5131 reflections	Δρ_max_ = 1.06 e Å^−^^3^
367 parameters	Δρ_min_ = −0.66 e Å^−^^3^
5 restraints	Extinction correction: none
Primary atom site location: structure-invariant direct methods	

### Special details

**Table:**

Geometry. All e.s.d.'s (except the e.s.d. in the dihedral angle between two l.s. planes) are estimated using the full covariance matrix. The cell e.s.d.'s are taken into account individually in the estimation of e.s.d.'s in distances, angles and torsion angles; correlations between e.s.d.'s in cell parameters are only used when they are defined by crystal symmetry. An approximate (isotropic) treatment of cell e.s.d.'s is used for estimating e.s.d.'s involving l.s. planes.
Refinement. Refinement of *F*^2^ against ALL reflections. The weighted *R*-factor *wR* and goodness of fit *S* are based on *F*^2^, conventional *R*-factors *R* are based on *F*, with *F* set to zero for negative *F*^2^. The threshold expression of *F*^2^ > σ(*F*^2^) is used only for calculating *R*-factors(gt) *etc*. and is not relevant to the choice of reflections for refinement. *R*-factors based on *F*^2^ are statistically about twice as large as those based on *F*, and *R*- factors based on ALL data will be even larger.

### Fractional atomic coordinates and isotropic or equivalent isotropic displacement parameters (Å^2^)

**Table:**

	*x*	*y*	*z*	*U*_iso_*/*U*_eq_	Occ. (<1)
Mn1	0.5000	0.0000	0.5000	0.04145 (18)	
Mn2	0.7500	0.7500	0.54180 (3)	0.04268 (18)	
N1	0.53293 (9)	0.2142 (3)	0.50330 (12)	0.0505 (6)	
N2	0.58426 (10)	0.3776 (3)	0.48747 (15)	0.0678 (8)	
H2N	0.6061	0.4234	0.4696	0.081*	
N3	0.54774 (8)	−0.0725 (3)	0.57988 (12)	0.0485 (6)	
N4	0.58594 (9)	−0.2212 (3)	0.64106 (13)	0.0560 (7)	
H4N	0.5943	−0.2989	0.6591	0.067*	
N5	0.44833 (8)	0.0685 (3)	0.57579 (12)	0.0520 (6)	
N6	0.41219 (10)	0.0705 (3)	0.66934 (14)	0.0634 (8)	
H6N	0.4064	0.0574	0.7099	0.076*	
N7	0.71986 (9)	0.9721 (3)	0.54032 (13)	0.0562 (7)	
N8	0.69433 (10)	1.1818 (3)	0.56814 (17)	0.0687 (8)	
H8N	0.6860	1.2514	0.5923	0.082*	
O1	0.65926 (8)	0.0707 (2)	0.13121 (11)	0.0653 (6)	
O2	0.60909 (8)	−0.0251 (2)	0.19895 (10)	0.0606 (6)	
O3	0.69820 (10)	0.6144 (3)	0.35534 (12)	0.0948 (10)	
O4	0.65304 (8)	0.5144 (3)	0.42532 (10)	0.0657 (7)	
O1W	0.68093 (7)	0.6472 (2)	0.53876 (9)	0.0512 (5)	
H1A	0.6718	0.6126	0.4983	0.077*	
H1B	0.6757	0.5844	0.5679	0.077*	
O2W	0.7500	0.7500	0.43813 (13)	0.0597 (8)	
H2A	0.7658	0.8011	0.4125	0.090*	
C1	0.56746 (13)	0.2553 (4)	0.46893 (18)	0.0675 (10)	
H1	0.5792	0.2031	0.4345	0.081*	
C2	0.56039 (16)	0.4160 (5)	0.5397 (3)	0.0967 (15)	
H2	0.5651	0.4958	0.5653	0.116*	
C3	0.52808 (14)	0.3172 (4)	0.5483 (2)	0.0817 (12)	
H3	0.5059	0.3196	0.5804	0.098*	
C4	0.55176 (11)	−0.2037 (3)	0.60003 (16)	0.0558 (8)	
H4	0.5327	−0.2765	0.5869	0.067*	
C5	0.60497 (13)	−0.0920 (4)	0.6489 (2)	0.0779 (11)	
H5	0.6295	−0.0697	0.6753	0.094*	
C6	0.58146 (12)	−0.0023 (4)	0.6111 (2)	0.0712 (10)	
H6	0.5874	0.0938	0.6069	0.085*	
C7	0.44988 (11)	0.0350 (4)	0.63811 (16)	0.0580 (8)	
H7	0.4745	−0.0083	0.6579	0.070*	
C8	0.38476 (13)	0.1312 (5)	0.6245 (2)	0.0810 (12)	
H8	0.3560	0.1675	0.6318	0.097*	
C9	0.40725 (12)	0.1289 (4)	0.56727 (19)	0.0736 (11)	
H9	0.3962	0.1636	0.5280	0.088*	
C10	0.71022 (13)	1.0575 (4)	0.58866 (18)	0.0663 (9)	
H10	0.7140	1.0341	0.6323	0.080*	
C11	0.69375 (15)	1.1776 (5)	0.5028 (2)	0.0842 (12)	
H11	0.6844	1.2493	0.4749	0.101*	
C12	0.70935 (15)	1.0492 (4)	0.4858 (2)	0.0819 (12)	
H12	0.7125	1.0174	0.4432	0.098*	
C20	0.65821 (10)	0.4142 (3)	0.31930 (14)	0.0459 (7)	
C21	0.69273 (10)	0.3442 (4)	0.28942 (16)	0.0609 (9)	
H21	0.7224	0.3681	0.2995	0.073*	
C22	0.68436 (10)	0.2363 (4)	0.24350 (16)	0.0576 (9)	
H22	0.7086	0.1916	0.2235	0.069*	
C23	0.64154 (9)	0.1962 (3)	0.22789 (13)	0.0438 (6)	
C24	0.60419 (9)	0.2703 (3)	0.25621 (13)	0.0406 (6)	
C25	0.55847 (10)	0.2406 (3)	0.23935 (15)	0.0506 (7)	
H25	0.5523	0.1678	0.2103	0.061*	
C26	0.52392 (10)	0.3157 (4)	0.26466 (16)	0.0619 (9)	
H26	0.4944	0.2929	0.2533	0.074*	
C27	0.53196 (11)	0.4280 (4)	0.30797 (17)	0.0636 (9)	
H27	0.5079	0.4804	0.3243	0.076*	
C28	0.57517 (11)	0.4599 (3)	0.32607 (16)	0.0536 (8)	
H28	0.5802	0.5339	0.3550	0.064*	
C29	0.61259 (9)	0.3822 (3)	0.30145 (13)	0.0408 (6)	
C30	0.67030 (11)	0.5228 (3)	0.37099 (15)	0.0523 (8)	
C31	0.63602 (10)	0.0715 (3)	0.18193 (14)	0.0482 (7)	
N9	0.7500	0.7500	0.64847 (19)	0.0789 (9)	
N10	0.7681 (2)	0.7068 (7)	0.7487 (3)	0.0789 (9)	0.50
H10A	0.7842	0.7041	0.7834	0.095*	0.50
C13	0.78353 (16)	0.7509 (9)	0.6908 (2)	0.0789 (9)	0.50
H13	0.8131	0.7777	0.6819	0.095*	0.50
C14	0.7240 (2)	0.6669 (9)	0.7465 (3)	0.0789 (9)	0.50
H14	0.7053	0.6331	0.7794	0.095*	0.50
C15	0.71499 (17)	0.6904 (9)	0.6824 (3)	0.0789 (9)	0.50
H15	0.6874	0.6679	0.6633	0.095*	0.50

### Atomic displacement parameters (Å^2^)

**Table:**

	*U*^11^	*U*^22^	*U*^33^	*U*^12^	*U*^13^	*U*^23^
Mn1	0.0469 (3)	0.0377 (4)	0.0398 (3)	0.0007 (3)	0.0026 (2)	0.0047 (3)
Mn2	0.0572 (4)	0.0383 (4)	0.0326 (3)	−0.0074 (3)	0.000	0.000
N1	0.0579 (15)	0.0432 (15)	0.0503 (15)	−0.0055 (12)	−0.0004 (12)	0.0041 (11)
N2	0.0686 (18)	0.0568 (19)	0.078 (2)	−0.0232 (15)	−0.0048 (16)	0.0047 (16)
N3	0.0525 (14)	0.0432 (15)	0.0498 (14)	0.0048 (11)	−0.0035 (11)	0.0045 (12)
N4	0.0600 (15)	0.0519 (17)	0.0560 (16)	0.0127 (13)	−0.0053 (13)	0.0106 (13)
N5	0.0556 (15)	0.0499 (15)	0.0506 (15)	0.0027 (12)	0.0090 (12)	0.0023 (12)
N6	0.0746 (18)	0.0633 (19)	0.0524 (16)	−0.0028 (15)	0.0200 (14)	−0.0069 (14)
N7	0.0631 (16)	0.0419 (15)	0.0637 (17)	−0.0015 (13)	0.0016 (13)	−0.0002 (13)
N8	0.0686 (18)	0.0457 (17)	0.092 (2)	0.0059 (14)	0.0175 (17)	−0.0035 (16)
O1	0.0891 (16)	0.0559 (14)	0.0509 (13)	−0.0123 (12)	0.0252 (12)	−0.0153 (11)
O2	0.0784 (15)	0.0518 (14)	0.0516 (13)	−0.0218 (12)	0.0107 (11)	−0.0151 (10)
O3	0.129 (2)	0.102 (2)	0.0540 (14)	−0.077 (2)	0.0049 (14)	−0.0151 (14)
O4	0.0837 (16)	0.0693 (16)	0.0441 (13)	−0.0292 (13)	0.0057 (11)	−0.0172 (11)
O1W	0.0607 (12)	0.0498 (13)	0.0431 (11)	−0.0133 (10)	0.0037 (9)	0.0033 (9)
O2W	0.082 (2)	0.065 (2)	0.0327 (14)	−0.0347 (17)	0.000	0.000
C1	0.080 (2)	0.056 (2)	0.066 (2)	−0.0206 (19)	0.0131 (19)	−0.0044 (18)
C2	0.106 (3)	0.059 (3)	0.125 (4)	−0.019 (2)	0.012 (3)	−0.031 (3)
C3	0.086 (3)	0.062 (2)	0.097 (3)	−0.013 (2)	0.020 (2)	−0.025 (2)
C4	0.0620 (19)	0.0449 (19)	0.061 (2)	−0.0002 (15)	−0.0067 (16)	0.0078 (15)
C5	0.076 (2)	0.061 (2)	0.097 (3)	0.003 (2)	−0.040 (2)	0.003 (2)
C6	0.073 (2)	0.047 (2)	0.093 (3)	−0.0006 (17)	−0.028 (2)	0.0060 (19)
C7	0.0611 (19)	0.065 (2)	0.0476 (18)	0.0006 (16)	0.0089 (15)	−0.0035 (16)
C8	0.067 (2)	0.091 (3)	0.085 (3)	0.021 (2)	0.025 (2)	0.000 (2)
C9	0.070 (2)	0.083 (3)	0.069 (2)	0.025 (2)	0.0110 (18)	0.013 (2)
C10	0.087 (3)	0.045 (2)	0.067 (2)	0.0017 (18)	0.0127 (19)	−0.0015 (17)
C11	0.097 (3)	0.065 (3)	0.091 (3)	0.026 (2)	−0.004 (2)	0.012 (2)
C12	0.112 (3)	0.067 (3)	0.067 (2)	0.024 (2)	−0.012 (2)	0.003 (2)
C20	0.0513 (16)	0.0452 (17)	0.0412 (15)	−0.0115 (13)	−0.0016 (12)	−0.0077 (13)
C21	0.0435 (16)	0.075 (2)	0.064 (2)	−0.0141 (16)	0.0012 (15)	−0.0213 (18)
C22	0.0449 (16)	0.068 (2)	0.0602 (19)	−0.0042 (15)	0.0068 (14)	−0.0221 (17)
C23	0.0480 (15)	0.0454 (16)	0.0381 (14)	−0.0061 (13)	0.0019 (12)	−0.0092 (13)
C24	0.0451 (14)	0.0417 (16)	0.0349 (14)	−0.0035 (12)	−0.0039 (11)	−0.0011 (12)
C25	0.0477 (16)	0.0563 (19)	0.0478 (16)	−0.0046 (14)	−0.0075 (13)	−0.0063 (15)
C26	0.0437 (17)	0.081 (2)	0.061 (2)	0.0022 (16)	−0.0135 (15)	−0.0003 (19)
C27	0.0534 (18)	0.073 (2)	0.065 (2)	0.0204 (17)	−0.0030 (16)	−0.0065 (18)
C28	0.0619 (19)	0.0467 (18)	0.0523 (18)	0.0082 (15)	−0.0063 (15)	−0.0082 (14)
C29	0.0477 (15)	0.0372 (15)	0.0376 (14)	−0.0013 (12)	−0.0026 (11)	−0.0007 (12)
C30	0.0614 (18)	0.0502 (19)	0.0452 (18)	−0.0130 (15)	−0.0087 (14)	−0.0049 (14)
C31	0.0528 (16)	0.0450 (18)	0.0468 (17)	−0.0051 (14)	−0.0005 (13)	−0.0089 (13)
N9	0.101 (2)	0.087 (2)	0.0486 (13)	0.0064 (19)	0.000	0.000
N10	0.101 (2)	0.087 (2)	0.0486 (13)	0.0064 (19)	0.000	0.000
C13	0.101 (2)	0.087 (2)	0.0486 (13)	0.0064 (19)	0.000	0.000
C14	0.101 (2)	0.087 (2)	0.0486 (13)	0.0064 (19)	0.000	0.000
C15	0.101 (2)	0.087 (2)	0.0486 (13)	0.0064 (19)	0.000	0.000

### Geometric parameters (Å, °)

**Table:**

Mn1—N1^i^	2.250 (3)	C4—H4	0.9300
Mn1—N1	2.250 (3)	C5—C6	1.344 (5)
Mn1—N3^i^	2.271 (2)	C5—H5	0.9300
Mn1—N3	2.271 (2)	C6—H6	0.9300
Mn1—N5^i^	2.276 (2)	C7—H7	0.9300
Mn1—N5	2.276 (2)	C8—C9	1.351 (5)
Mn2—N7^ii^	2.283 (3)	C8—H8	0.9300
Mn2—N7	2.283 (3)	C9—H9	0.9300
Mn2—N9	2.190 (4)	C10—H10	0.9300
Mn2—O1W^ii^	2.265 (2)	C11—C12	1.346 (6)
Mn2—O1W	2.265 (2)	C11—H11	0.9300
Mn2—O2W	2.129 (3)	C12—H12	0.9300
N1—C1	1.302 (4)	C20—C21	1.363 (4)
N1—C3	1.352 (4)	C20—C29	1.432 (4)
N2—C1	1.316 (4)	C20—C30	1.521 (4)
N2—C2	1.335 (5)	C21—C22	1.412 (4)
N2—H2N	0.8600	C21—H21	0.9300
N3—C4	1.314 (4)	C22—C23	1.362 (4)
N3—C6	1.360 (4)	C22—H22	0.9300
N4—C4	1.327 (4)	C23—C24	1.433 (4)
N4—C5	1.356 (5)	C23—C31	1.520 (4)
N4—H4N	0.8600	C24—C25	1.425 (4)
N5—C7	1.319 (4)	C24—C29	1.430 (4)
N5—C9	1.355 (4)	C25—C26	1.350 (4)
N6—C7	1.330 (4)	C25—H25	0.9300
N6—C8	1.355 (5)	C26—C27	1.406 (5)
N6—H6N	0.8600	C26—H26	0.9300
N7—C10	1.312 (4)	C27—C28	1.366 (5)
N7—C12	1.372 (5)	C27—H27	0.9300
N8—C10	1.335 (5)	C28—C29	1.423 (4)
N8—C11	1.342 (5)	C28—H28	0.9300
N8—H8N	0.8600	N9—C13	1.3195 (10)
O1—C31	1.248 (3)	N9—C13^ii^	1.3195 (10)
O2—C31	1.262 (4)	N9—C15^ii^	1.3703 (10)
O3—C30	1.239 (4)	N9—C15	1.3703 (10)
O4—C30	1.230 (4)	N10—C13	1.3400 (10)
O1W—H1A	0.9331	N10—C14	1.3597 (11)
O1W—H1B	0.8576	N10—H10A	0.8600
O2W—H2A	0.8542	C13—H13	0.9300
C1—H1	0.9300	C14—C15	1.3603 (11)
C2—C3	1.349 (6)	C14—H14	0.9300
C2—H2	0.9300	C15—H15	0.9300
C3—H3	0.9300		
			
N1^i^—Mn1—N1	180.00 (12)	C5—C6—N3	110.1 (3)
N1^i^—Mn1—N3^i^	88.89 (9)	C5—C6—H6	124.9
N1—Mn1—N3^i^	91.11 (9)	N3—C6—H6	124.9
N1^i^—Mn1—N3	91.11 (9)	N5—C7—N6	112.2 (3)
N1—Mn1—N3	88.89 (9)	N5—C7—H7	123.9
N3^i^—Mn1—N3	180.00 (8)	N6—C7—H7	123.9
N1^i^—Mn1—N5^i^	90.80 (9)	C9—C8—N6	106.8 (3)
N1—Mn1—N5^i^	89.20 (9)	C9—C8—H8	126.6
N3^i^—Mn1—N5^i^	90.60 (9)	N6—C8—H8	126.6
N3—Mn1—N5^i^	89.40 (9)	C8—C9—N5	109.7 (3)
N1^i^—Mn1—N5	89.20 (9)	C8—C9—H9	125.2
N1—Mn1—N5	90.80 (9)	N5—C9—H9	125.2
N3^i^—Mn1—N5	89.40 (9)	N7—C10—N8	112.4 (3)
N3—Mn1—N5	90.60 (9)	N7—C10—H10	123.8
N5^i^—Mn1—N5	180.00 (9)	N8—C10—H10	123.8
O2W—Mn2—N9	180.000 (1)	N8—C11—C12	106.4 (4)
O2W—Mn2—O1W^ii^	88.42 (5)	N8—C11—H11	126.8
N9—Mn2—O1W^ii^	91.58 (5)	C12—C11—H11	126.8
O2W—Mn2—O1W	88.42 (5)	C11—C12—N7	110.2 (4)
N9—Mn2—O1W	91.58 (5)	C11—C12—H12	124.9
O1W^ii^—Mn2—O1W	176.85 (10)	N7—C12—H12	124.9
O2W—Mn2—N7^ii^	89.24 (7)	C21—C20—C29	119.3 (3)
N9—Mn2—N7^ii^	90.76 (7)	C21—C20—C30	117.8 (3)
O1W^ii^—Mn2—N7^ii^	92.42 (9)	C29—C20—C30	122.9 (3)
O1W—Mn2—N7^ii^	87.54 (9)	C20—C21—C22	121.3 (3)
O2W—Mn2—N7	89.24 (7)	C20—C21—H21	119.3
N9—Mn2—N7	90.76 (7)	C22—C21—H21	119.3
O1W^ii^—Mn2—N7	87.54 (9)	C23—C22—C21	121.5 (3)
O1W—Mn2—N7	92.42 (9)	C23—C22—H22	119.3
N7^ii^—Mn2—N7	178.48 (14)	C21—C22—H22	119.3
C1—N1—C3	103.8 (3)	C22—C23—C24	119.1 (3)
C1—N1—Mn1	126.4 (2)	C22—C23—C31	117.6 (3)
C3—N1—Mn1	128.6 (2)	C24—C23—C31	123.3 (2)
C1—N2—C2	105.8 (3)	C25—C24—C29	118.0 (3)
C1—N2—H2N	127.1	C25—C24—C23	122.5 (3)
C2—N2—H2N	127.1	C29—C24—C23	119.4 (2)
C4—N3—C6	104.3 (3)	C26—C25—C24	121.5 (3)
C4—N3—Mn1	124.7 (2)	C26—C25—H25	119.3
C6—N3—Mn1	130.6 (2)	C24—C25—H25	119.3
C4—N4—C5	106.3 (3)	C25—C26—C27	120.9 (3)
C4—N4—H4N	126.9	C25—C26—H26	119.6
C5—N4—H4N	126.9	C27—C26—H26	119.6
C7—N5—C9	104.9 (3)	C28—C27—C26	119.9 (3)
C7—N5—Mn1	124.8 (2)	C28—C27—H27	120.1
C9—N5—Mn1	129.4 (2)	C26—C27—H27	120.1
C7—N6—C8	106.4 (3)	C27—C28—C29	121.2 (3)
C7—N6—H6N	126.8	C27—C28—H28	119.4
C8—N6—H6N	126.8	C29—C28—H28	119.4
C10—N7—C12	103.9 (3)	C28—C29—C24	118.6 (3)
C10—N7—Mn2	130.0 (2)	C28—C29—C20	122.3 (3)
C12—N7—Mn2	126.1 (2)	C24—C29—C20	119.1 (2)
C10—N8—C11	107.1 (3)	O4—C30—O3	123.9 (3)
C10—N8—H8N	126.5	O4—C30—C20	119.4 (3)
C11—N8—H8N	126.5	O3—C30—C20	116.7 (3)
Mn2—O1W—H1A	116.0	O1—C31—O2	125.1 (3)
Mn2—O1W—H1B	116.3	O1—C31—C23	117.6 (3)
H1A—O1W—H1B	109.0	O2—C31—C23	117.3 (3)
Mn2—O2W—H2A	128.0	C13—N9—C15	103.7 (4)
N1—C1—N2	113.7 (3)	C13—N9—Mn2	131.2 (3)
N1—C1—H1	123.1	C15—N9—Mn2	120.6 (3)
N2—C1—H1	123.1	C13—N10—C14	112.6 (6)
N2—C2—C3	107.0 (4)	C13—N10—H10A	123.7
N2—C2—H2	126.5	C14—N10—H10A	123.7
C3—C2—H2	126.5	N9—C13—N10	109.0 (5)
C2—C3—N1	109.5 (4)	N9—C13—H13	125.5
C2—C3—H3	125.2	N10—C13—H13	125.5
N1—C3—H3	125.2	N10—C14—C15	100.0 (6)
N3—C4—N4	112.7 (3)	N10—C14—H14	130.0
N3—C4—H4	123.6	C15—C14—H14	130.0
N4—C4—H4	123.6	C14—C15—N9	114.3 (5)
C6—C5—N4	106.6 (3)	C14—C15—H15	122.9
C6—C5—H5	126.7	N9—C15—H15	122.9
N4—C5—H5	126.7		

Symmetry codes: (i) −*x*+1, −*y*, −*z*+1; (ii) −*x*+3/2, −*y*+3/2, *z*.

### Hydrogen-bond geometry (Å, °)

**Table:**

*D*—H···*A*	*D*—H	H···*A*	*D*···*A*	*D*—H···*A*
O1W—H1A···O4	0.93	1.85	2.772 (3)	170
O1W—H1B···O1^iii^	0.86	2.02	2.875 (3)	175
O2W—H2A···O3^ii^	0.85	1.78	2.624 (3)	172
N2—H2N···O4	0.86	1.87	2.730 (4)	176
N4—H4N···O2^iv^	0.86	1.91	2.765 (3)	177
N6—H6N···O2^i^	0.86	1.95	2.810 (4)	178
N8—H8N···O1^v^	0.86	2.02	2.870 (4)	167
N10—H10A···O3^vi^	0.86	1.78	2.560 (7)	150

Symmetry codes: (iii) *x*, −*y*+1/2, *z*+1/2; (ii) −*x*+3/2, −*y*+3/2, *z*; (iv) *x*, −*y*−1/2, *z*+1/2; (i) −*x*+1, −*y*, −*z*+1; (v) *x*, −*y*+3/2, *z*+1/2; (vi) −*x*+3/2, *y*, *z*+1/2.

## References

[bb1] Altomare, A., Cascarano, G., Giacovazzo, C. & Guagliardi, A. (1993). *J. Appl. Cryst.* **26**, 343–350.

[bb2] Derissen, J. L., Timmermans, C. & Schoone, J. C. (1979). *Cryst. Struct. Commun.* **8**, 533–536.

[bb3] Farrugia, L. J. (1997). *J. Appl. Cryst.* **30**, 565.

[bb4] Farrugia, L. J. (1999). *J. Appl. Cryst.* **32**, 837–838.

[bb5] Higashi, T. (1995). *ABSCOR* Rigaku Corporation, Tokyo, Japan.

[bb6] Liu, B.-X., Su, J.-R. & Xu, D.-J. (2004). *Acta Cryst.* C**60**, m183–m185. 10.1107/S0108270104005529 15071212

[bb7] Rigaku (1998). *PROCESS-AUTO* Rigaku Corporation, Tokyo, Japan.

[bb8] Rigaku/MSC (2002). *CrystalStructure* Rigaku/MSC, The Woodlands, Texas, USA.

[bb9] Sheldrick, G. M. (2008). *Acta Cryst.* A**64**, 112–122. 10.1107/S0108767307043930 18156677

[bb10] Su, J.-R. & Xu, D.-J. (2004). *J. Coord. Chem.* **57**, 223–229.

